# Across a macro-ecological gradient forest competition is strongest at the most productive sites

**DOI:** 10.3389/fpls.2014.00260

**Published:** 2014-06-05

**Authors:** Lynda D. Prior, David M. J. S. Bowman

**Affiliations:** School of Biological Sciences, University of TasmaniaHobart, TAS, Australia

**Keywords:** climate, diameter increment, tree size, competition, basal area, *Eucalyptus*

## Abstract

We tested the hypothesis that the effect of forest basal area on tree growth interacts with macro-ecological gradients of primary productivity, using a large dataset of eucalypt tree growth collected across temperate and sub- tropical mesic Australia. To do this, we derived an index of inter-tree competition based on stand basal area (stand BA) relative to the climatically determined potential basal area. Using linear mixed effects modeling, we found that the main effects of climatic productivity, tree size, and competition explained 26.5% of the deviance in individual tree growth, but adding interactions to the model could explain a further 8.9%. The effect of competition on growth interacts with the gradient of climatic productivity, with negligible effect of competition in low productivity environments, but marked negative effects at the most productive sites. We also found a positive interaction between tree size and stand BA, which was most pronounced in the most productive sites. We interpret these patterns as reflecting intense competition for light amongst maturing trees on more productive sites, and below ground moisture limitation at low productivity sites, which results in open stands with little competition for light. These trends are consistent with the life history and stand development of eucalypt forests: in cool moist environments, light is the most limiting resource, resulting in size-asymmetric competition, while in hot, low rainfall environments are open forests with little competition for light but where the amount of tree regeneration is limited by water availability.

## Introduction

Tree growth rates are influenced by many factors including climate and edaphic properties, tree size, and the competitive environment (Gómez-Aparicio et al., 2011; Craine and Dybzinski, 2013). Ranking the relative importance of these factors presents formidable practical challenges given the complexity of the interactions and the spatial and temporal scales involved in working with trees. Macro-ecological studies using extensive networks of permanent forest plots offer the opportunity to investigate trends in growth responses over large spatial and temporal scales, and across a wide range of species and environmental conditions to discern the relative effects of species, tree size, competition, climate, and edaphic factors (Canham et al., 2006; Kunstler et al., 2011; Gómez-Aparicio et al., 2011).

We have assembled a dataset of tree growth measurements from 2409 plots in temperate Australia to undertake macro-ecological research into tree growth. We have found that eucalypt growth is positively correlated with water availability but negatively related to mean annual temperatures in excess of 11°C (Bowman et al., 2014). We have also demonstrated that eucalypt growth is subject to a strong negative interaction between temperature and tree size (Prior and Bowman, 2014). Our data present an opportunity to use statistical modeling to assess the relative influence on eucalypt growth of inter-tree competition, climate, and tree size across a wide productivity gradient.

Competition is the process by which two or more individuals acquire resources from a common, potentially limiting supply (Craine and Dybzinski, 2013). Grime (1977) theorized that the importance of competition in unproductive habitats is small relative to the impact of the abiotic constraints on plant growth. We therefore expected that growth would be most sensitive to competition in the most productive environments, manifest in a negative climatic productivity by competition interaction.

Competition can be broadly separated into above ground competition for light, and below ground competition for water and nutrients. Above ground competition is often considered to be asymmetric, because larger trees are able to capture a disproportionately large share of light through shading of smaller trees (Schwinning and Weiner, 1998; Craine and Dybzinski, 2013). On the other hand, competition for water and nutrients is generally assumed to be more symmetric, with the soil volume depleted of these resources being approximately proportional to plant size (Schwinning and Weiner, 1998; Craine and Dybzinski, 2013). The intensity and degree of size-asymmetry in competition falls on a continuum, and is likely to change along a gradient of site productivity (Schwinning and Weiner, 1998; Van Breugel et al., 2012).

Stand basal area (stand BA), which incorporates the number of trees in a stand and their diameters, is a frequently-used index of inter-tree competition in both local scale and regional studies (Weiskittel et al., 2011). It often performs similarly to more complex, distance-dependent measures of competition (Nyström and Kexi, 1997; Kiernan et al., 2008; Stage and Ledermann, 2008). However, because local and regional effects of BA can offset each other across macro-ecological gradients, studies of inter-tree competition and tree growth must account for differences in site productivity. For example, in northern Australia, a strong negative effect of stand BA on eucalypt growth was detected in a local-scale study (Prior et al., 2006), but not in a regional study that spanned a 500 mm rainfall gradient (Murphy et al., 2010). In the local-scale study, high stand BA at a local scale was associated with increased competition and therefore reductions in tree growth, but at a regional scale higher BA was associated with improved site quality and thus was correlated with increased tree growth.

Weiskittel et al. (2011) noted that stand BA is not a true measure of competition unless it is combined with some measure of stand development. However, the term “stand development” implies a stand is single-aged, and frequently regenerating, multi-aged stands may also be stocked below their climatic potential as a result of disturbances such as fire, storm damage or disease. So in addition to stand BA, we developed an index of inter-tree competition that accounts for actual stand BA relative to site productivity, irrespective of whether a stand is single-aged or multi-aged. We termed this “relative basal area” (RBA), defined as the ratio of actual stand BA to climatically determined potential stand BA, square-root transformed. We reasoned that below-ground competition should be more closely related to water availability and thus RBA, but that above-ground competition should be more closely related to absolute stand BA than to RBA, because incident light flux is not directly related to climatic productivity (water availability and temperature). In other words, a particular stand BA value may represent similar competition for light in high and low productivity environments, but represent greater competition for soil water (and a higher RBA) in low productivity environments.

Here, we investigate the relative importance of climate, tree size, and competition for growth rates of eucalypts at a continental scale, comparing stand BA and RBA as proxies for competition. We also use interactions between these factors to analyze how the intensity of competition, and the degree of size-asymmetry in competition, varied across the climatic productivity gradient, reasoning that the larger the asymmetry, the larger the positive interaction between size and stand BA, We discuss these patterns of competition across productivity gradients in relation to the ecology of eucalypts.

## Materials and methods

### Tree size and growth measurements

**Figure 1:**
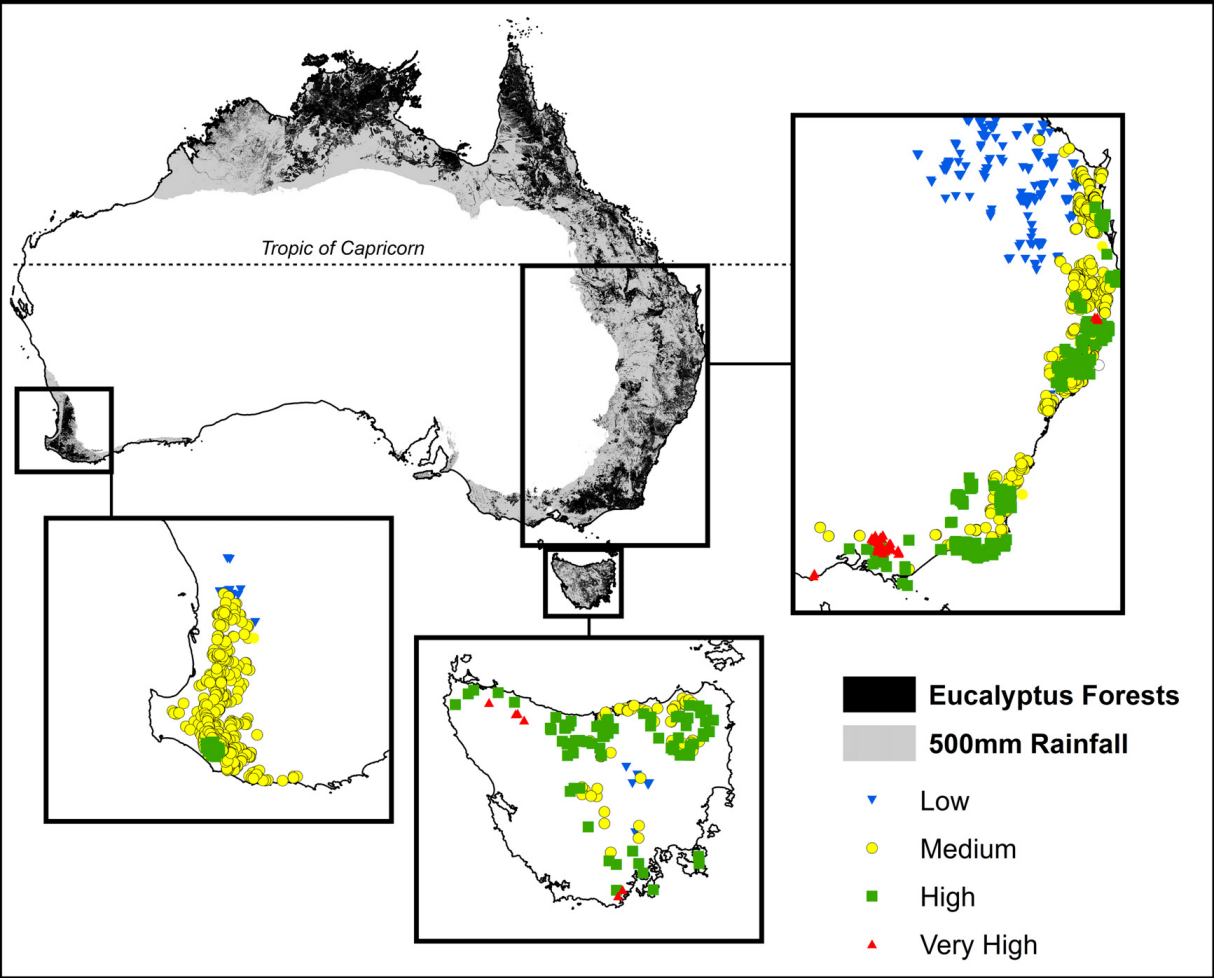
**Location of plots showing climatic productivity categories, derived from maximum temperature of the warmest month and the ratio of precipitation to evaporation.** These plots were all located outside of the tropics in areas receiving >500 mm mean annual precipitation (shown in gray). Climatic and growth characteristics of these plots are summarized in Table 1.

Permanent growth plots have been established to monitor tree growth in temperate forests by Australian state government forestry organizations since the 1930s (Bowman et al., 2014). Our study focused on permanent plots located in temperate mesic eucalypt forests, defined as forests outside the tropics that receive >500 mm mean annual precipitation (Figure 1). Our plots spanned a gradient in mean annual precipitation of 558–2105 mm and mean annual temperature of 6.4–22.4°C. The most productive forests in the study region are located in cool, moist areas of south-eastern Australia, and are among those with the highest biomass on earth (Keith et al., 2009) (Figure 1).

The plots are naturally regenerating, often with a pulse of recruitment following disturbance such as wildfire. Some forests have been thinned or logged at various times, which provides a spectrum of sizes, ages, and intensity of competition across the continent. Plots were re-measured after thinning, so the reduction in stand BA is incorporated in our dataset, and our analysis captures its effects on growth. These forests are generally multi-aged, but the age of most trees is not known. Approximately one-third of plots contained only one species of eucalypt >10 cm diameter.

The tree growth data consisted of repeated measurements of the diameter at breast height (DBH) of individually identified trees within marked plots of known area and location with measurement intervals averaging 4.0 years (range 1–44 years) (Bowman et al., 2014). In most cases, all trees >10 cm DBH within a plot were measured, but in some plots only large trees (e.g., >50 cm DBH) were measured over the entire plot, and smaller trees measured in sub-plots of known area. Diameter increments were annualized. For our analyses, we used only eucalypt growth data complying with the following conditions: measurement interval ≥1 year; plot size ≥100 m^2^; stand BA 10–100 m^2^ ha^−1^; eucalypts with DBH from 10–150 cm, and diameter increments from −0.5–2.5 cm year^−1^. This filtering was done to avoid gross measurement/recording error, very high stand BAs arising from a very large tree on a small plot, and plots that had very recently been clear-felled. After filtering, the dataset comprised records from 2409 plots, and 499,161 tree–intervals and >100 species or subspecies.

In most cases, the spatial configuration of trees within plots was not specified, so it was not possible to compare the effect of competition from larger neighbors with that of competition from all neighbors (e.g., Coomes and Allen, 2007). However, the degree of the size-asymmetry of competition can be inferred from the interactive effect of tree size and competition on growth rates, given that in any stand, large trees will have fewer larger neighbors than the small trees have.

### Explanatory variables

**Figure 2:**
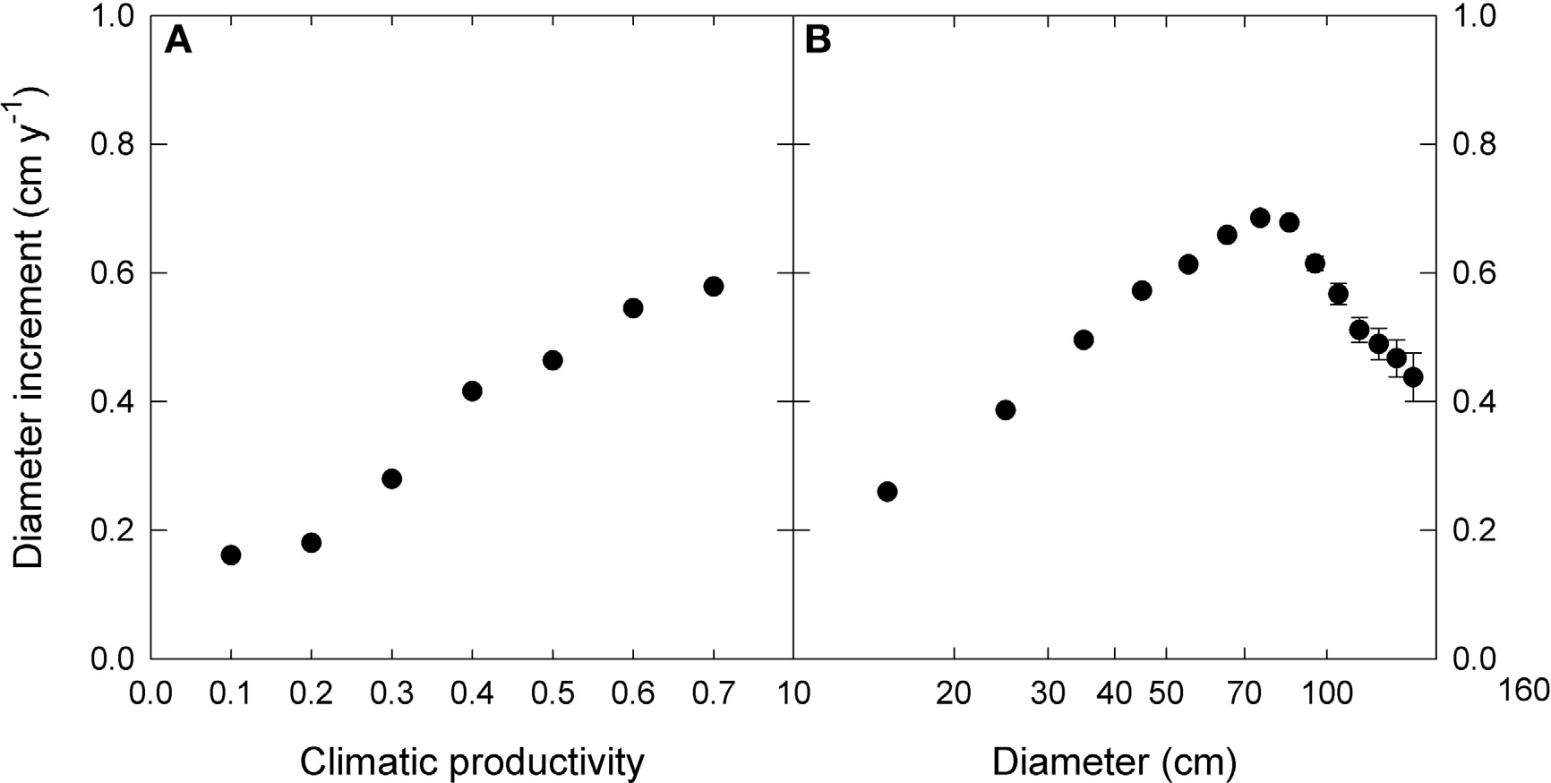
**The relationship between diameter increment and (A) climatic productivity index and (B) initial diameter.** Note the logarithmic scale for diameter. For presentation, data were grouped into 0.1- climatic productivity index classes and 10 cm—diameter classes. Standard errors are shown where larger than the symbols.

We used linear mixed effects modeling to describe the growth effects of climate, tree size and inter-tree competition, as well as their interactions. In order to do this, for each of the three factors we derived the following single measures to which growth displayed an approximately linear response (Figure 2).

#### Climatic productivity

Climatic data were obtained from the WORLDCLIM dataset (Hijmans et al., 2005) for the BIOCLIM variables mean annual precipitation (P) and mean annual temperature (T). Pan evaporation (E) was derived from ANUCLIM 6.1 (Australian National University, Canberra).

**Figure 3:**
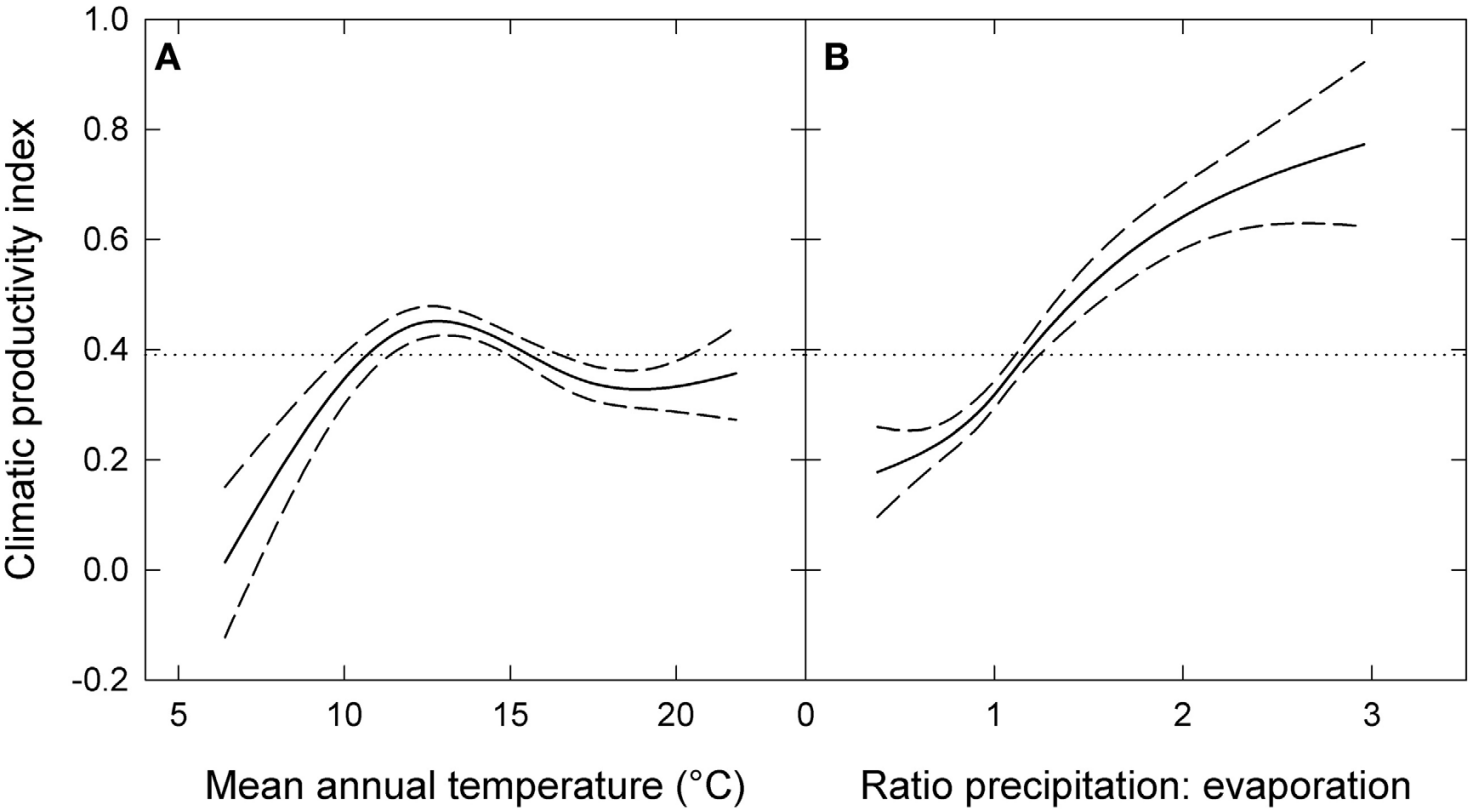
**Climatic productivity index as a function of (A) mean annual temperature and (B) the ratio of precipitation to evaporation.** The index was based on a generalized additive model describing eucalypt growth (in cm year^−1^) in relation to these variables. Dashed lines indicate 95% confidence intervals and the dotted lines show mean growth rate for the entire dataset. Responses to each variable were calculated by holding the other variable constant at its mean value.

The response of eucalypt growth to climate variables is complex and non-linear (Bowman et al., 2014). We therefore derived an index of climatic productivity to which eucalypt growth showed an approximately linear response. This was based on a generalized additive model of eucalypt diameter growth (in cm year^−1^) in relation to T (mean annual temperature) and P:E (the ratio Precipitation: Evaporation, an index of water availability) (Bowman et al., 2014). (We used the model containing T rather than maximum temperature of the warmest month because this better describes the growth response at the cool end of the data range.) This climatic productivity index model was based on plot growth means, and explained 24% of the deviance in growth rates (Figure 3). The R package mgcv (v.1.6-2) was used for the generalized additive modeling. Low, medium, high and very high climatic productivity plots were considered to be those with a climatic productivity index of ≤0.2, 0.2–0.4, 0.4–0.6, and >0.6, respectively. The geographic patterning of climatic productivity is shown in Figure 1, and temperature, rainfall, water availability and stand characteristics of the climatic productivity classes are summarized in Table 1.

**Table 1:** **Summary of climatic conditions, stand basal area, and tree size in the four Climatic Productivity Categories, defined according to growth rate predicted from mean annual temperature and the ratio of precipitation to evaporation**.

	**Units**	**Climatic productivity category**							
		**Low**		**Medium**		**High**		**Very high**	
		**Mean**	***SE***	**Mean**	***SE***	**Mean**	***SE***	**Mean**	***SE***
Climatic productivity index (predicted growth)	Cm year^−1^	0.15	0.0001	0.31	0.0001	0.49	0.0001	0.66	0.0001
Diameter increment	Cm year^−1^	0.16	0.001	0.28	0.001	0.48	0.001	0.56	0.001
Mean annual temperature	°C	19.4	0.009	16.6	0.007	12.9	0.005	11.2	0.002
Mean maximum temperature of the warmest month	°C	31.7	0.009	27.4	0.006	24.7	0.004	24.2	0.003
Mean annual precipitation	mm	750	0.7	1107	0.5	1168	0.5	1493	0.5
Ratio precipitation: evaporation	Dimensionless ratio	0.54	0.001	1.00	0.0004	1.38	0.001	2.03	0.001
Initial stem diameter	cm	27.6	0.10	31.6	0.05	29.3	0.04	37.7	0.05
Stand basal area	m^2^ ha^−1^	15.3	0.04	27.3	0.03	39.6	0.04	47.0	0.05
Stand basal area—90th percentile	m^2^ ha^−1^	26.8	0.03	40.6	0.03	57.3	0.02	69.3	0.02
Relative basal area	Dimensionless ratio	0.75	0.0007	0.81	0.0004	0.82	0.0004	0.81	0.004

#### Tree size

Initial DBH was used as the measure of tree size. It was log-transformed to normalize the data. We also calculated the coefficient of variation (CV) of the log-transformed DBH of all trees in a plot the first time it was measured, to examine the relationship between variability in tree size and climatic productivity.

#### Inter-tree competition

The stand BA of each plot (an indicator of inter-tree competition) was calculated by summing the cross-sectional area of each tree stem, then dividing by the ground area. Where sub-plots were used, stand BA was calculated for each size class individually, then summed to give total stand BA for the plot (trees of all species were included in the stand BA calculations).

Stand BA was correlated with T and P:E (Table 2 and Bowman et al., 2014). To express stand BA relative to the climatically-determined potential for each plot, we first calculated the 90th percentile of stand BA (BA90) as a function of T and P:E, using the R quantreg package:

BA.90=90.6−3.55*T+9.11*P:E

**Table 2:** **Correlation matrix for growth, tree size, climatic, and competition variables**.

	**Diameter increment**	**l.DBH**	**CV-l.DBH**	**Stand BA**	**BA.90**	**RBA**	***P***	***T***	**Max warm**	**Min cold**	***E***	***P:E***	**Climatic productivity**
Diam incr	1.00	0.31	−0.45	−0.13	0.26	−0.33	0.16	−0.24	−0.22	−0.19	−0.27	0.25	0.28
l.DBH	0.31	1.00	0.03	0.11	0.16	0.03	0.23	−0.13	−0.09	−0.11	−0.10	0.21	0.17
CV−l.DBH	−0.45	0.03	1.00	0.03	−0.36	0.24	−0.17	0.31	0.27	0.23	0.37	−0.39	−0.45
Stand BA	−0.13	0.11	0.03	1.00	0.55	0.79	0.26	−0.55	−0.49	−0.45	−0.56	0.45	0.53
BA.90	0.26	0.16	−0.36	0.55	1.00	−0.03	0.49	−0.98	−0.85	−0.84	−0.94	0.86	0.88
RBA	−0.33	0.03	0.24	0.79	−0.03	1.00	0.00	0.02	−0.02	0.06	−0.02	−0.03	0.03
P	0.16	0.23	−0.17	0.26	0.49	0.00	1.00	−0.33	−0.37	−0.19	−0.35	0.82	0.70
T	−0.24	−0.13	0.31	−0.55	−0.98	0.02	−0.33	1.00	0.85	0.87	0.94	−0.74	−0.80
Max warm	−0.22	−0.09	0.27	−0.49	−0.85	−0.02	−0.37	0.85	1.00	0.68	0.87	−0.67	−0.73
Min cold	−0.19	−0.11	0.23	−0.45	−0.84	0.06	−0.19	0.87	0.68	1.00	0.78	−0.60	−0.62
E	−0.27	−0.10	0.37	−0.56	−0.94	−0.02	−0.35	0.94	0.87	0.78	1.00	−0.77	−0.85
P:E	0.25	0.21	−0.39	0.45	0.86	−0.03	0.82	−0.74	−0.67	−0.60	−0.77	1.00	0.91
Climatic productivity	0.28	0.17	−0.45	0.53	0.88	0.03	0.70	−0.80	−0.73	−0.62	−0.85	0.91	1.00

Variables shown are Diam.incr., annual diameter increment, l.DBH, log-transformed diameter at breast height, CV-l.DBH, coefficient of variation in l.DBH, stand BA, stand basal area, BA.90, climatically determined 90th percentile basal area, RBA, relative basal area, P, mean annual precipitation, T, mean annual temperature), Max Warm, average daily maximum temperature of the warmest month, Min Cold, average daily minimum temperature of the coldest month, E, evaporation, P:E, the ratio of P to E, and the climatic productivity index. n = 499,161 tree—intervals.

RBA was then calculated as the square root of (stand BA/BA.90). The square root transformation was used to normalize the data. We note that stand BA is also influenced by soil fertility and physical characteristics, and that by definition, 10% of plots will exceed the 90th percentile. Thus the RBA of some plots was > 1.0.

### Data analyses

The magnitude and importance of the effects of climate, tree size and competition, and their various interactions, was investigated using linear mixed effects modeling and model selection based on a robust form of Akaike's information criterion (AICc), a model selection index favoring both model fit and model simplicity (Burnham and Anderson, 2002). To establish the importance of the interactions, together and individually, we compared the model containing the three main effects and all two-factor interactions with models containing the three main effects but only one interaction and the model with three main effects but no interactions. Finally, we added the three-factor interaction (climatic productivity × size × competition) to the model with the three two-factor interactions to assess whether size-asymmetric competition was more important in the most productive environments. Stand BA was used as the proxy for competition in one set of candidate models, with the analysis was repeated using RBA. Plot was a random effect in all the models to account for the spatial autocorrelation of individual tree growth. We present results of the analysis that used data from only those trees <70 cm diameter, for which the growth response is approximately log-linear (Prior and Bowman, 2014). The results were very similar, but less deviance was explained, when the full dataset was used. We also investigated whether diameter increment should be log-transformed in the analyses (with an offset for negative values). However, these linear mixed effects models were problematic, with positive log likelihoods and negative % deviance explained. We found that the direction and importance of the effects were similar to the models using untransformed diameter increment, giving us confidence in our conclusions based on untransformed diameter increment.

The sensitivity of individual species to competition was assessed from the slope of the relationship between diameter increment and stand BA for the 30 species with >1600 observations. We present this analysis for small trees only (<30 cm diameter), because these are most sensitive to competition and are well-represented for all major species in our dataset. Results were similar but noisier when trees of all sizes were used.

The statistical software R was used for all analyses (R Development Core Team, 2013). The R package lme4 was used for the linear mixed effects modeling.

## Results

Tree diameter, diameter increment, and stand BA increased, but variability in tree diameter decreased, with increasing climatic productivity (Table 2).

### Ranking of climate, tree size, and competition

Our analysis showed that tree size and competitive effects had a much stronger influence than climate on individual tree growth. Climatic productivity on its own explained only 0.2% of the deviance in the growth of individual eucalypts over the climatic gradient, which spanned more than 1500 mm in mean annual rainfall and 16°C in mean annual temperature. [The correlation of diameter increment with climatic productivity was higher in the raw data (*r*^2^ = 0.08; Table 2); some of this was apparently subsumed by the random effect, plot]. Local site and individual tree factors appeared to have a stronger influence than climate on tree growth, as RBA alone explained 9% of the deviance and tree diameter explained 12% (Table 3). Combining the three factors climatic productivity, tree diameter and RBA improved the explanatory power of the modeling. The additive model containing the three factors climatic productivity, initial diameter and RBA explained 26% of the deviance in the growth data, more than the sum of the deviance explained by the individual factors (~21%; Table 3).

**Table 3:** **Comparison of linear mixed effects models describing individual tree growth**.

**Model**	***K***	**Comp = stand basal area**		**Comp = RBA**	
		**Delta AIC_**c**_**	**Deviance expl. (%)**	**Delta AIC_**c**_**	**Deviance expl. (%)**
Comp ^*^ Climatic productivity ^*^ l.DBH	8	0	34.47	0	35.37
Comp ^*^ Climatic productivity + Comp ^*^ l.DBH + Climatic productivity ^*^ l.DBH	7	11	34.47	576	35.23
Comp + Climatic productivity ^*^ l.DBH	5	3018	33.73	5562	34
Comp ^*^ l.DBH + Climatic productivity	5	15166	30.74	32742	27.33
Comp ^*^ Climatic productivity + l.DBH	5	34836	25.91	35055	26.76
Comp + Climatic productivity + l.DBH	4	35033	25.86	36023	26.52
l.DBH	2	91555	11.98	95226	11.98
Comp	2	107787	7.99	107792	8.89
Climatic productivity	2	139696	0.16	143368	0.16
Intercept only	1	140329	NA	144001	NA

Explanatory variables were Comp, competition, climatic productivity index, l.DBH, log-transformed diameter at breast height, and the stated interactions. The proxy used for competition was either stand basal area or RBA, relative basal area. Plot was a random effect in all models. The analyses were performed on only those trees <70 cm diameter, where the growth response is approximately linear and positive. The global model, which included all three two-factor interactions, was clearly the best, given that models with a delta AIC > 10 have essentially no statistical support (Burnham and Anderson, 2002). K is the number of parameters in the model and percent deviance explained is relative to the null (intercept only) model. (n = 475,821).

### RBA vs. BA as competition proxies

**Figure 4:**
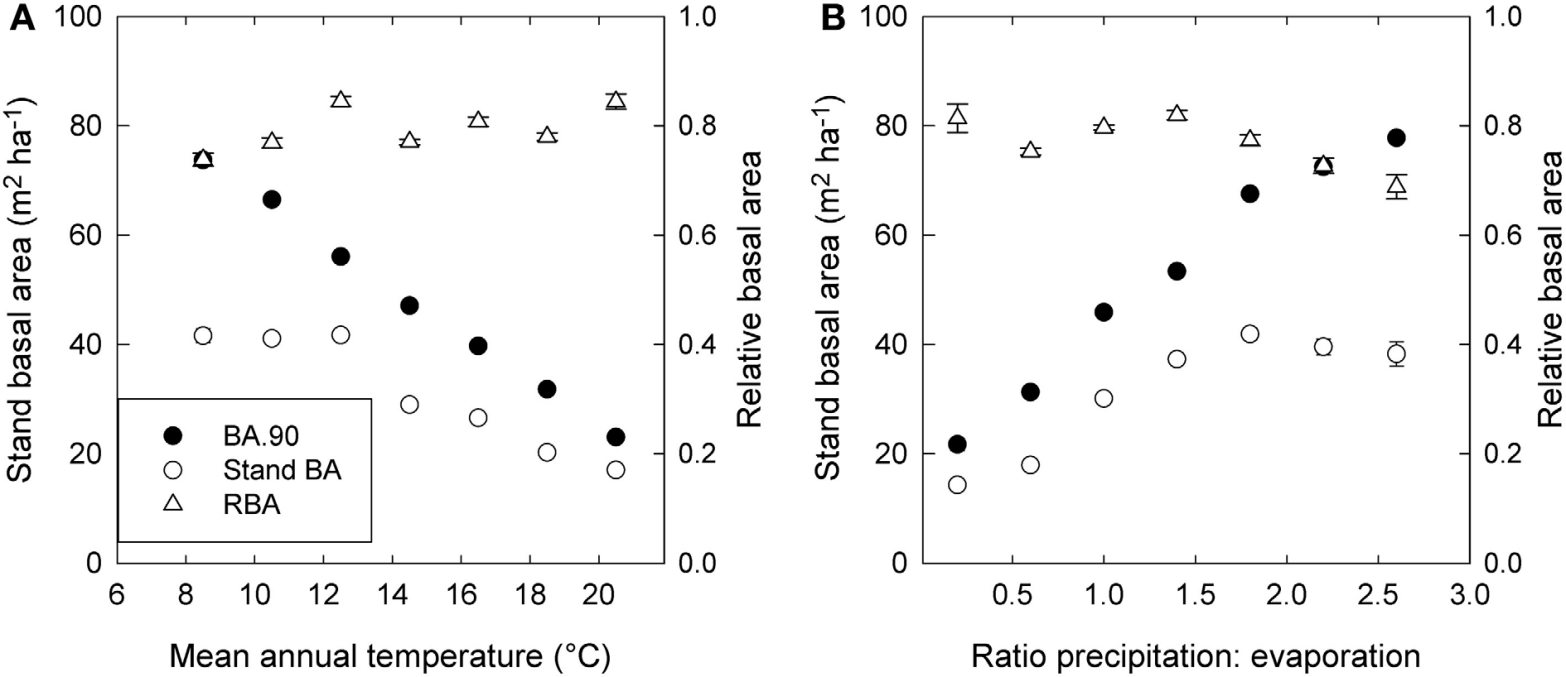
**Trends in stand basal area (Stand BA), the 90th percentile of basal area (BA.90) and relative basal area (RBA) in relation to (A) mean annual temperature, and (B) the ratio of precipitation to evaporation (P.E).** Standard errors are shown where larger than the symbol. Values are based on plot means, and for presentation are grouped into 2°—temperature classes and 0.4—P.E classes.

Stand BA was moderately correlated with climatic variables (*r*^2 = 0.07^ to 0.31), especially those relating to temperature and evaporation (Table 2; Figure 4). By contrast, RBA was essentially uncorrelated with any climatic variables (Table 2), supporting its use as a measure of stand development that was independent of climate.

**Figure 5:**
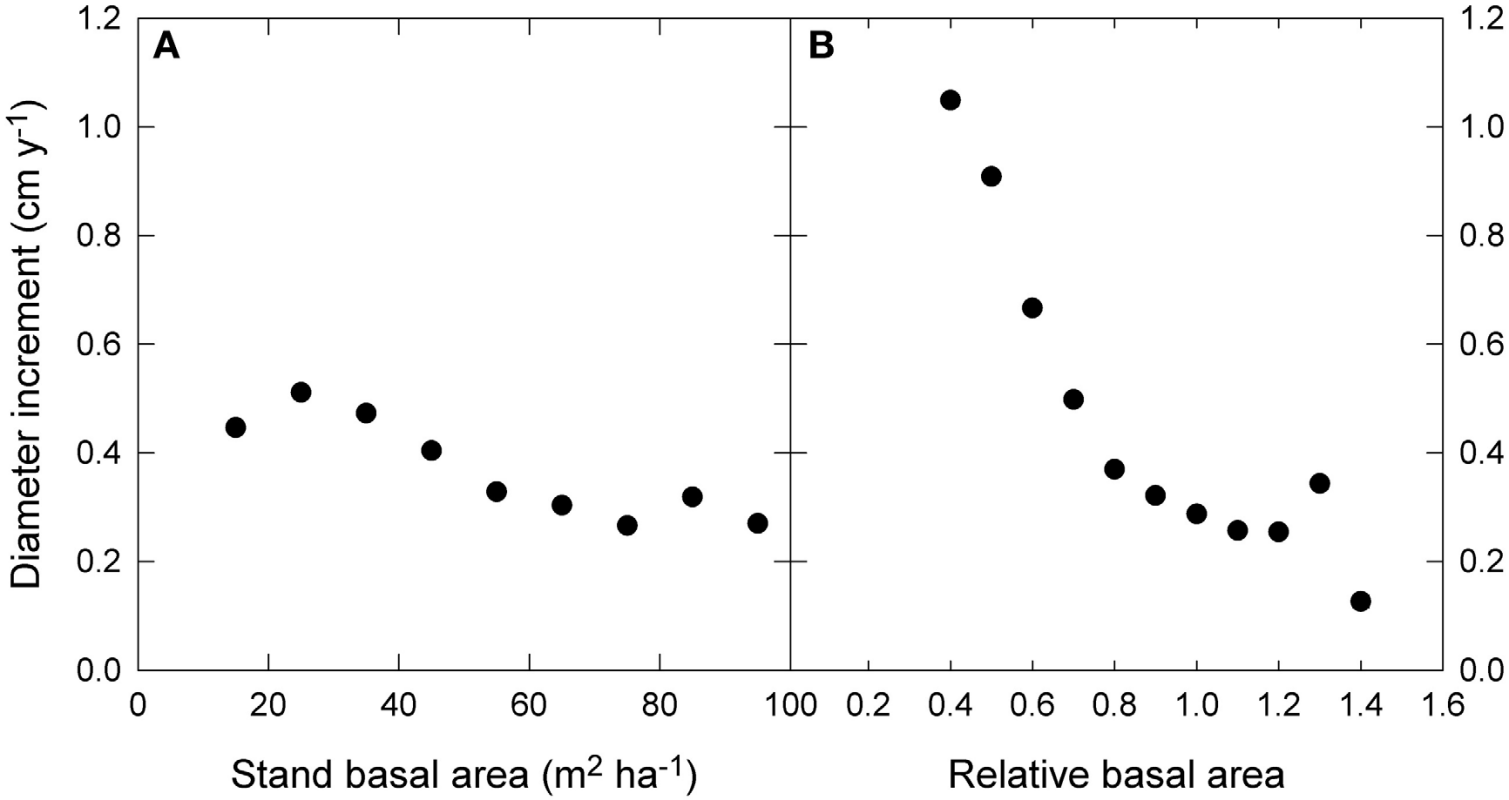
**Relationship between diameter growth and (A) stand basal area, and (B) relative basal area.** Relative basal area was calculated as square root (stand basal area/the 90th percentile of basal area). The 90th percentile of basal area was estimated by quantile regression of growth as a function of water availability and mean annual temperature. Standard error bars are shown where larger than the symbol.

**Figure 6:**
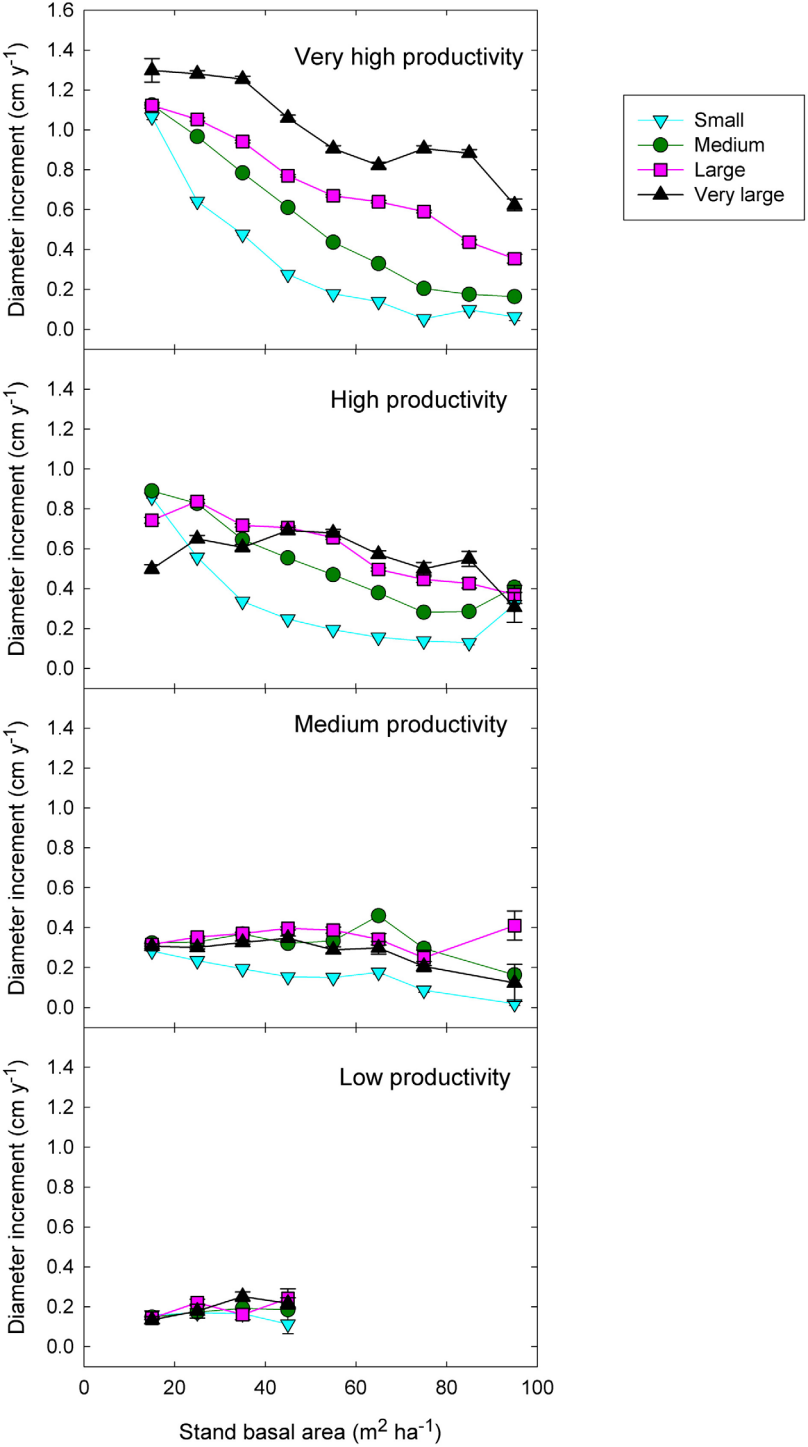
**Eucalypt diameter growth in relation to stand basal area, tree size, and climatic productivity category.** (This is analogous to the left-hand graphs in Figure 7, except that stand BA has been substituted for relative basal area).

The raw data showed only a slight decline in growth with increasing stand BA, but a much stronger decline with RBA (Figure 5). Modeled growth responses were similar for stand BA and RBA (Figure 6), but models with RBA generally had better explanatory power, so we focus on describing responses to RBA (Table 3).

Although models with RBA were generally superior to those with stand BA, there was an exception regarding the interaction between competition and tree size (an indicator of asymmetric competition). The (stand BA^*^DBH + climatic productivity) model explained substantially more deviance than the (RBA^*^DBH + climatic productivity) model (31% *cf* 27%) (Table 3).

### Intensity and size-asymmetry of competition in relation to climatic productivity

**Figure 7:**
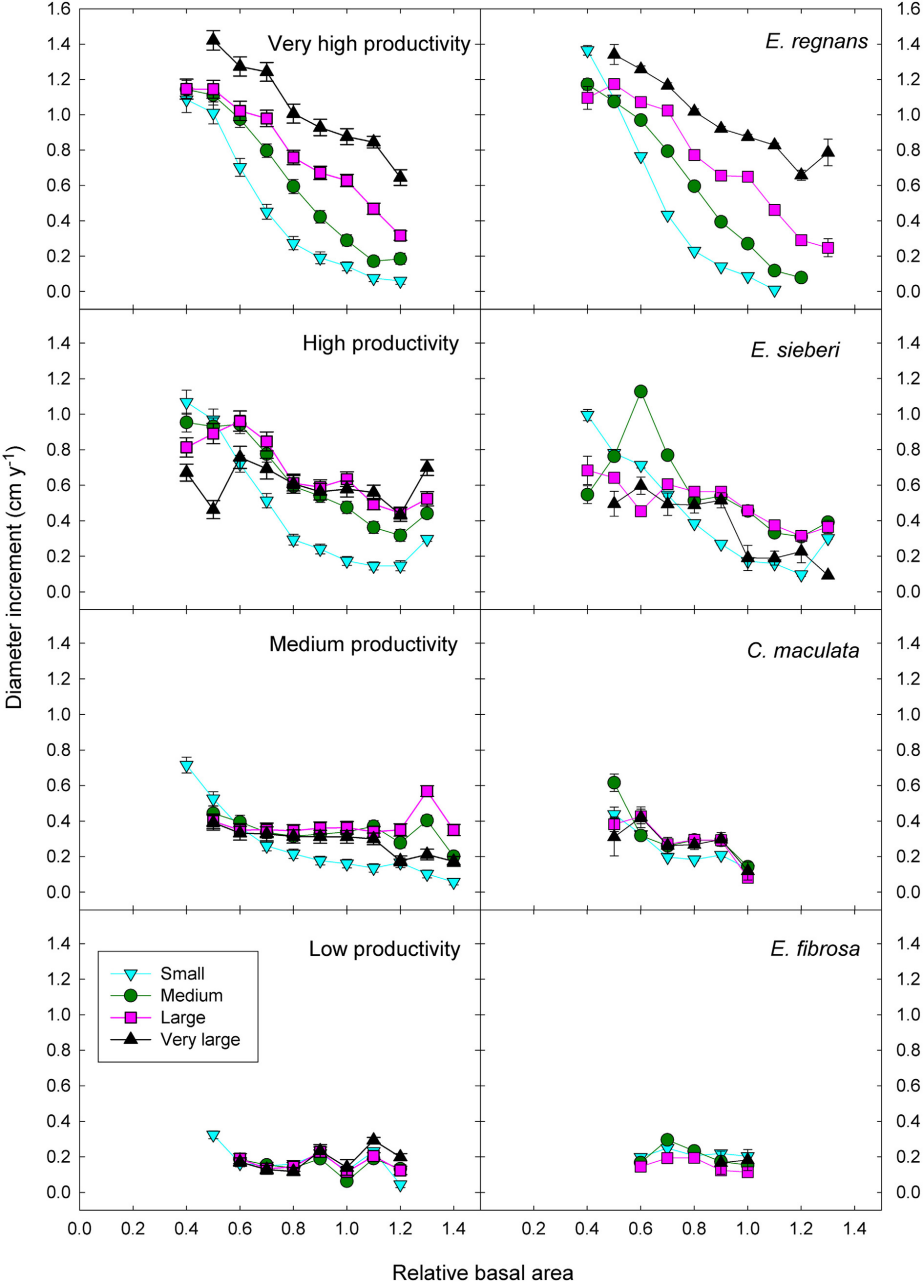
**Eucalypt diameter growth in relation to relative basal area, tree size, and climatic productivity category.** Data for a representative species growing in the climatic productivity category is presented in the right hand column. There was statistical support for all interactions. Standard errors are shown where larger than the symbol.

Adding the three two-factor interactions boosted the deviance explained to 35% (Table 3). There was strong statistical support for all two-factor interactions, given that all models with interactions outranked the model with only the three additive terms (Table 3). The most important interaction was the positive climatic productivity by DBH interaction, whereby small trees are relatively insensitive to climate but large trees grow much faster in cool moist climates than in hotter, drier ones (Figure 7; Tables 3, 4).

**Table 4:** **Summary of effects on growth of climatic productivity, tree size, and our proxies for competition (stand BA and RBA), and their interactions, from linear mixed effects modeling**.

**Effect**	**Direction**	**Competition proxy (% deviance expl.)**		**Interpretation**
		**Stand BA**	**RBA**	
Climatic productivity	+			Best growth in most productive climates
Tree size	+			Large trees grow fastest
Competition	−	8.0	**8.9**	Growth declines with increasing competition, and is more closely linked to competition relative to its climatic potential than to absolute stand BA
Climatic productivity × tree size	+			Large trees grow especially fast in the most productive climates, and are especially affected by unfavorable climates
Climatic productivity × competition	−	0.05	**0.24**	Adverse effect of competition on growth is greatest in the most productive climates. Best correlated with RBA, which reflects competition relative to climatic potential
Tree size × competition	+	**4.9**	0.8	Asymmetric competition—adverse effect of competition on growth is weaker for large trees than for small ones, and more marked for absolute stand BA, which provides a better measure of shading than does RBA
Climatic productivity × tree size × competition	+	<0.01	**0.14**	Asymmetric competition is most pronounced in the most productive climates. Best correlated with RBA, which reflects competition relative to climatic potential

The direction of effects was the same for both competition proxies, but the magnitude differed, as shown by the % deviance explained (relative to the simpler model without that term). The better proxy for each term is shown in bold. There was statistical support for all effects listed (Table 3). Coefficients and standard errors of the key RBA models are listed in Table 5.

**Table 5:** **Coefficients and associated standard errors for the global model describing eucalypt growth in terms of climatic productivity, tree size, and competition, and their interactions**.

**Term**	**Global model**		**Model with two-factor interactions**		**Additive model**	
	**Estimate**	***SE***	**Estimate**	***SE***	**Estimate**	***SE***
Intercept	0.71	0.06	2.08	0.03	0.18	0.02
Climatic productivity	1.23	0.13	−1.70	0.06	0.82	0.04
l.DBH	−0.22	0.04	−1.17	0.02	0.77	0.003
RBA	0.18	0.08	−1.55	0.03	−1.54	0.01
Climatic productivity × l.DBH	1.00	0.09	3.01	0.02		
Climatic productivity × RBA	−6.10	0.16	−2.40	0.04		
l.DBH × RBA	−0.38	0.05	0.81	0.02		
Climatic productivity × l.DBH × RBA	2.54	0.11				

Adding interaction terms can sometimes reverse the sign of coefficients (For example, DBH is positively correlated with growth, but when the climatic productivity by DBH interaction is added to the model, the sign of the main DBH effect becomes negative), so coefficients from simpler models are presented to indicate the direction of the main effects and two-factor interactions. The coefficients presented here are from models using only trees <70 cm diameter, for which the growth response is approximately log-linear; those based on the full dataset were similar. Tree diameter was log_10_-transformed.

In addition, there was a positive interaction between tree size and RBA, whereby growth of large trees was less affected than that of small ones by a high RBA (Figure 7; Tables 3, 4). There was also a negative interaction between climatic productivity and RBA, such that high RBA had the strongest adverse effect in the most productive environments (Figure 7). In other words, competition had the strongest negative effect on growth at the most productive sites: when RBA was high, growth rates of small trees at productive sites were as low as those in low productivity environments. By contrast, tree growth at low productivity sites was uniformly slow, showing little response to either tree diameter or to RBA (Figure 7).

As well as the two-factor interactions, there was statistical support for a small, positive three-factor interaction, which explained a further 0.2% of the deviance. Together, these interactions mean that at the most productive sites, large trees were able to grow particularly well, and were better able than small trees to cope with intense competition (Figure 7). We were thus able to demonstrate that across a large macro-climatic gradient, size-asymmetric competition was most pronounced at the more productive sites (Table 4).

### Competitive responses in individual species

**Figure 8:**
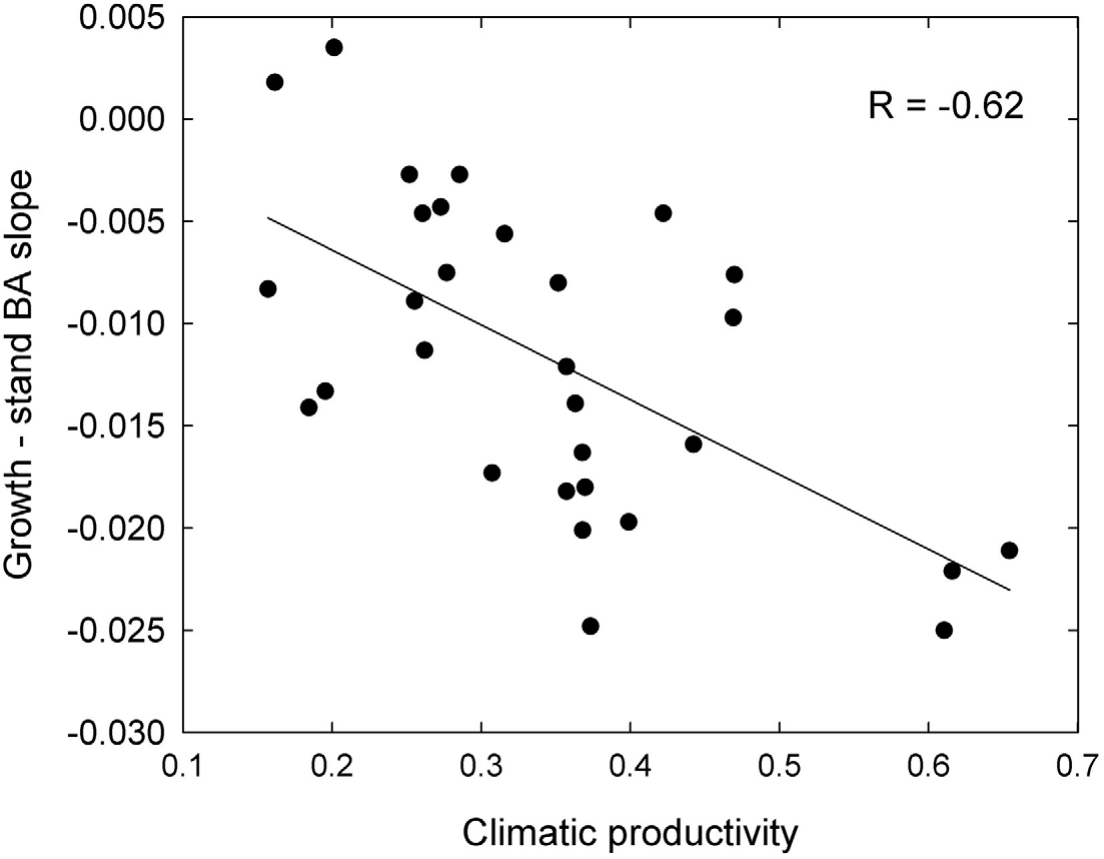
**Relationship between growth sensitivity to stand BA (growth—stand BA slope) and climatic productivity, for eucalypts <30 cm diameter**.

These responses to tree size and competition (both stand BA and RBA) were apparent in individual species growing in climates with contrasting productivity, as shown by the examples in Figure 7. For example, inter-tree competition and response to tree size were particularly pronounced in *Eucalyptus regnans*, which grows mostly in very high productivity environments (Figure 7). At the other extreme, *E. fibrosa*, found at sites with low to medium productivity, showed little growth response to either tree size or RBA (Figure 7). The stronger growth reductions in the most productive environments were apparent in responses to stand BA as well as to RBA. For instance, across the 30 species with >1600 observations, the slope of the growth-stand BA relationship was most negative in species growing in the most productive environments (Figure 8). In other words, for a given increase in stand BA there was a more severe growth reduction in the most productive environments.

## Discussion

### Relative importance of climate, tree size, and competition

We have analyzed a macro-ecological gradient spanning 1547 mm mean annual precipitation and 16°C mean annual temperature. On its own, climate had a surprisingly small effect on tree growth rates, explaining only 0.2% of the deviance in the data. RBA, as a proxy for competition, and tree size both had a much stronger effect, explaining 9 and 12% of the deviance respectively. This is similar to results from a Spanish study, which found that competition for water, light and nutrients from neighboring trees may exert an even stronger influence on tree growth than do tree size and climate (Gómez-Aparicio et al., 2011).

There were strong interactions amongst the three factors, indicating that favorable climates are especially advantageous for the growth of large trees (Prior and Bowman, 2014), but that inter-tree competition, and especially size-asymmetric competition, is most pronounced in these most productive climates, as discussed below.

### RBA vs. BA as a competition proxy

Using stand BA as a measure of competition is problematic when site quality varies, as in this study which spanned a very large climatic gradient. This is because high stand BA can be associated with either high productivity (positive relationship with growth) or with strong inter-tree competition (negative relationship with growth), and these two opposing effects can largely offset each other. Creating RBA had the desired effect of virtually removing any correlation with climatic variables, so that it reflected the degree of inter-tree competition relative to environmental productivity and, by extension, to stand development (Figure 4 and Table 2).

### Asymmetric and symmetric competition

We found that growth was more closely related to RBA than to stand BA, except with regards to the competition by tree size interaction, which is indicative of asymmetric competition. This interaction was much more pronounced when stand BA was used as the measure of competition than when RBA was used, whether alone or in combination with climate: adding this interaction to the 3-factor additive model explained an additional 4.8% of the deviance in the stand BA models compared with 0.8% for the RBA ones. Our analysis therefore suggests that absolute stand BA is more important than RBA in regards to asymmetric competition, whereby larger plants pre-empt directionally- supplied resources, most notably light (Schwinning and Weiner, 1998). We infer that RBA more closely reflects total competition relative to site quality, while stand BA is a better proxy for competition for light. Over the large climatic productivity gradient in this study, growth appeared more closely related to total competition, presumably because of the strong influence of water availability and temperature.

### Competition across productivity gradients

Grime (1977) theorized that competition is most important in productive habitats, and conversely, Bertness and Callaway (1994) postulated that positive biological interactions are more important in physically stressful habitats than in benign ones (the “stress-gradient hypothesis”). However, there have been only a few tests of these ideas for tree growth along large-scale bioclimatic gradients (Craine and Dybzinski, 2013): a New Zealand study showed competitive effects on tree growth declined with increasing altitude (Coomes and Allen, 2007); a French study that demonstrated the relative importance of competition declined with increasing abiotic stress (Kunstler et al., 2011); and a Spanish study contradicting this theory, finding that trees growing in low rainfall areas were more sensitive to competition than those at wetter sites (Gómez-Aparicio et al., 2011). Our results that competition relative to climatic potential, and asymmetric competition in particular (positive size by stand BA by climate interaction), are most pronounced in the most productive climates, provide additional support for Grime's (1977) theory, although the effects are relatively weak. The influence of climatic productivity on competition (manifest in a negative RBA by climate interaction) added only 0.24% to the deviance explained, and the three-factor interaction (suggesting that size asymmetric competition is also most pronounced in the high-productivity environments) boosted deviance explained by a further 0.14%. While mixed species forests may be more productive than pure stands, especially in poor sites (Pretzsch et al., 2013), this is unlikely to be a factor in our results, given the most productive eucalypt forests contained few species of eucalypts or other trees.

Our findings indicate that in the most productive environments, plants compete size asymmetrically early in stand development, as shown by the strong response to tree size when stand BA and RBA were low. The response to tree size was weaker as RBA increased, consistent with decreasing intensity of competition for light, because large trees are less likely to be overshadowed by taller neighbors (Coomes and Allen, 2007). By contrast, there was little effect of tree size in the low and medium productivity environments. Our results are therefore in line with other studies showing stronger competition for light in mesic than in xeric forests, because mesic forests have greater leaf area indices and capture more light (Grime, 1977; Coomes and Grubb, 2000). This has also been predicted to drive strong height growth, in keeping with the occurrence of some of the world's tallest forests in these environments (Coomes and Grubb, 2000; Tng et al., 2012).

### Eucalypt ecology and inter-tree competition

We expected that eucalypts, being shade-intolerant (Florence, 1996; Kariuki, 2008; Bond et al., 2012), would be strongly influenced by asymmetric competition (Kunstler et al., 2011). This was indeed the case, as shown by the positive interaction between tree size and competition, which indicates that competition for light suppresses growth of small trees. This is consistent with the ecology of these trees: there is very little eucalypt regeneration in undisturbed wet eucalypt forests, which occur in the most productive environments (Tng et al., 2012; Bowman et al., 2014). On the other hand, size symmetric competition is probably most prevalent in productive environments, because stands here contain trees of relatively uniform size.

Eucalypt regeneration is linked to stand to landscape scale disturbances, especially fire. Prevailing disturbance regimes, and thus the competitive relationships of eucalypts, are strongly influenced by climate (Ashton and Turner, 1979; Bowman and Kirkpatrick, 1986; Florence, 1996). For instance, eucalypt forests in the wettest areas typically exhibit massive regeneration following fire, resulting in even-aged cohorts. Initially, extremely dense regeneration competes strongly for light, but the stand rapidly thins as it matures, and competition for light amongst the remaining eucalypts diminishes. Mature wet forest typically consists of emergent eucalypts over an understory of rainforest trees, which are able to regenerate without disturbance (Tng et al., 2012). Our findings also accord with those of Canham et al. (2006), that competition for light has a strong influence on the growth of small trees, while trees of all sizes are affected by competition for nutrients.

In drier forests, tree size and stand density appear to be constrained by water availability. In these dry sclerophyll forests, adult eucalypts suppress juvenile eucalypts through competition for water (Rotheram, 1983; Bowman and Kirkpatrick, 1986), and during severe droughts, there is intense competition for water amongst adults, leading to canopy dieback and tree thinning (Fensham et al., 2009; Brouwers et al., 2013; Matusick et al., 2013). This is similar to other work showing that competition in a dry climate leads to widely-spaced dominants (Coomes and Grubb, 2000), and that competition diminishes in importance as abiotic stress increases (Kunstler et al., 2011).

The patterns described above, for eucalypts as a whole, are also evident for individual species (Figures 7, 8). For example, the world's tallest angiosperm, *Eucalyptus regnans*, grows in the highly productive cool moist forests of south-eastern Australia and is one of the most closely studied Australian tree species (Tng et al., 2012). Following fire, there is prolific regeneration from seed, leading to intense competition for light, which causes self-thinning and drives rapid height growth (Gilbert, 1959; Ashton and Turner, 1979). These trends are apparent in the rapid growth decrease in small trees in response to increasing competition (Figure 7). A similar strong growth response by young trees to the basal area of neighboring larger trees has been interpreted as showing competition for light is more important than below-ground competition in the initial successional phase of moist tropical forest (Van Breugel et al., 2012). By contrast, growth of *Eucalyptus fibrosa*, which grows in open forests in drier, warmer areas of eastern Australia, showed very little response to either tree size or RBA, probably because trees were smaller and more widely spaced (average stand BA was 15 m^2^ ha^−1^, cf. 44 m^2^ ha^−1^ for *E. regnans*), so that competition for light was much less intense (Figure 7).

To conclude, we have used our continental-scale dataset to demonstrate that inter-tree competition and tree size strongly modify the effects of climate on growth rates of individual eucalypts. Therefore, when examining growth rates over such a macro-climatic gradient, it is crucial to consider interactions between climate, tree size, and the competitive environment, in addition to the main effects. These interactions provided evidence of strong, size-asymmetric competition in the productive environments, but little effect of competition in the least productive environments. We also showed that when using stand BA as a proxy for competition across broad productivity gradients, it should be relativized to reflect site productivity. However, stand BA is a useful proxy for competition at the local scale, and as a measure of competition for light.

### Conflict of interest statement

The authors declare that the research was conducted in the absence of any commercial or financial relationships that could be construed as a potential conflict of interest.
